# Supplementary material for: Effect of Amyloid-β Monomers on Lipid Membrane Mechanical Parameters–Potential Implications for Mechanically Driven Neurodegeneration in Alzheimer’s Disease
Source: Int J Mol Sci. 2020 Dec 22;22(1):18. doi: 10.3390/ijms22010018 (PMC7792773; doi:10.3390/ijms22010018)
Supplement: Supplementary file 1 [file ijms-22-00018-s001.pdf]

Supporting Information: Effect of Amyloid-β Monomers on Lipid Membrane Mechanical Parameters—Potential Implications for Mechanically Driven Neurodegeneration in Alzheimer's Disease

Dominik Drabik [1,3,*], Grzegorz Chodaczek [2] and Sebastian Kraszewski [3]

[1] Laboratory of Cytobiochemistry, Faculty of Biotechnology, University of Wrocław, F. Joliot-Curie 14a, 50-383 Wrocław, Poland; Dominik.Drabik@UWr.edu.pl

[2] Łukasiewicz Research Network - PORT Polish Center for Technology Development, Stabłowicka 147, 54-066 Wrocław, Poland; Grzegorz.Chodaczek@port.lukasiewicz.gov.pl

[3] Department of Biomedical Engineering, Faculty of Fundamental Problems of Technology, Wrocław University of Science and Technology, Pl. Grunwaldzki 13, 50-377 Wrocław, Poland; Sebastian.Kraszewski@PWr.edu.pl

* Correspondence: Dominik.Drabik@UWr.edu.pl;

1. Bending rigidity measurements using flicker-noise spectroscopy

In Supplementary Figure S1 10 randomly selected vesicles are presented for each Aβ incorporated into POPC membrane obtained using the flicker-noise spectroscopy technique. Additionally, a control on purified POPC+DOTAP population was included. The populations were statistically different using both one-way ANOVA and Kruskal-Wallis, with following post-hoc Tukey test showing that POPC+DOTAP was significantly different from other populations, however all of Aβ incorporated membranes were not statistically significant from each other. The sample sizes of populations for Aβ-40, Aβ-42, and Aβ-40-TAMRA incorporated POPC membranes were 20, 16, and 17, respectively. The sample size of control was 10.

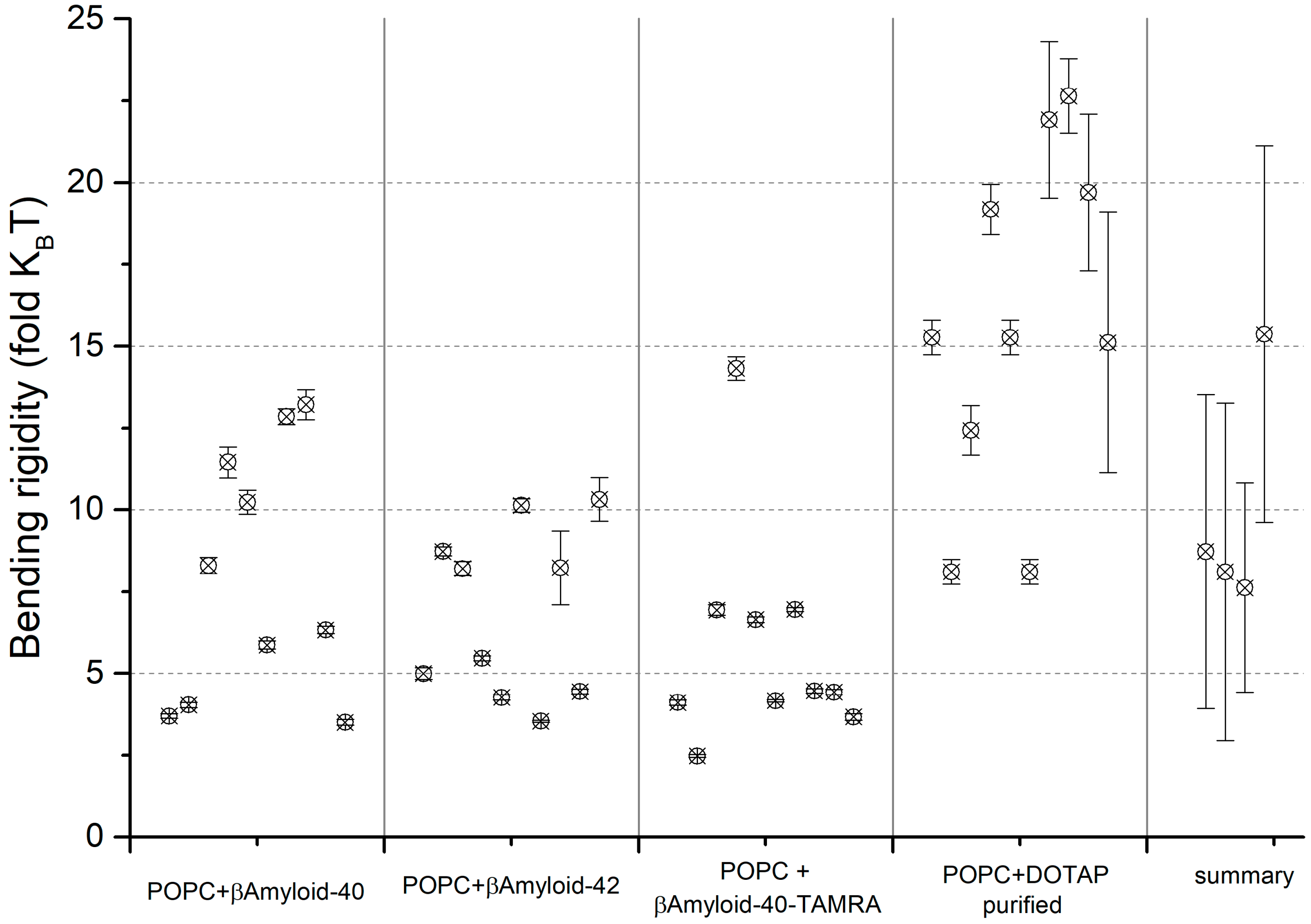

Figure S1. Bending rigidity obtained from 10 randomly selected vesicles for each of investigated βA peptides incorporated into POPC membrane. Additionally, purified POPC+DOTAP was presented as a control. Results were obtained from flicker-noise spectroscopy with average-based (ABV) approach.

2. Verification of βA incorporation in GUVs

In order to confirm the incorporation of βA peptides, a classical fluorescence visualization was used. GUVs were marked with Atto488-DOPE and incorporation of βA-40-TAMRA was investigated. Images with different excitation wavelength (488 nm for membrane and 530 nm for TAMRA) were recorded one after the other. Below in Supplementary Figures S2–S9 are images of the membrane (green), peptide (blue), and overlay fluorescence that confirm successful incorporation with the implemented method.

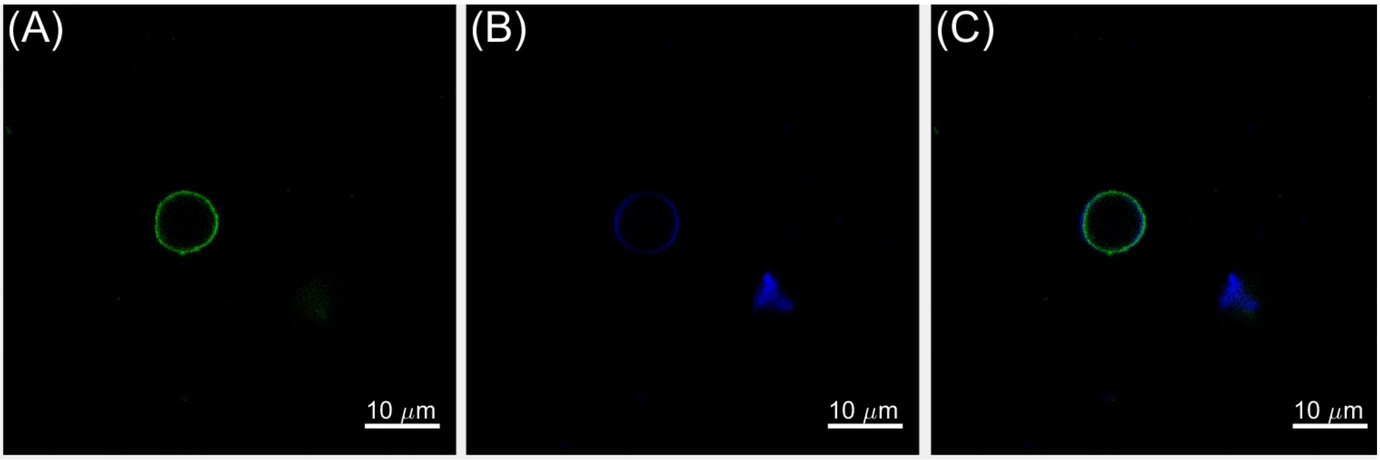

Figure S2. Fluorescence images of peptide location in lipid GUV. (A) Lipid membrane location determined using lipid probe (green). (B) Peptide location using TAMRA probe. (C) Overlay of the images.

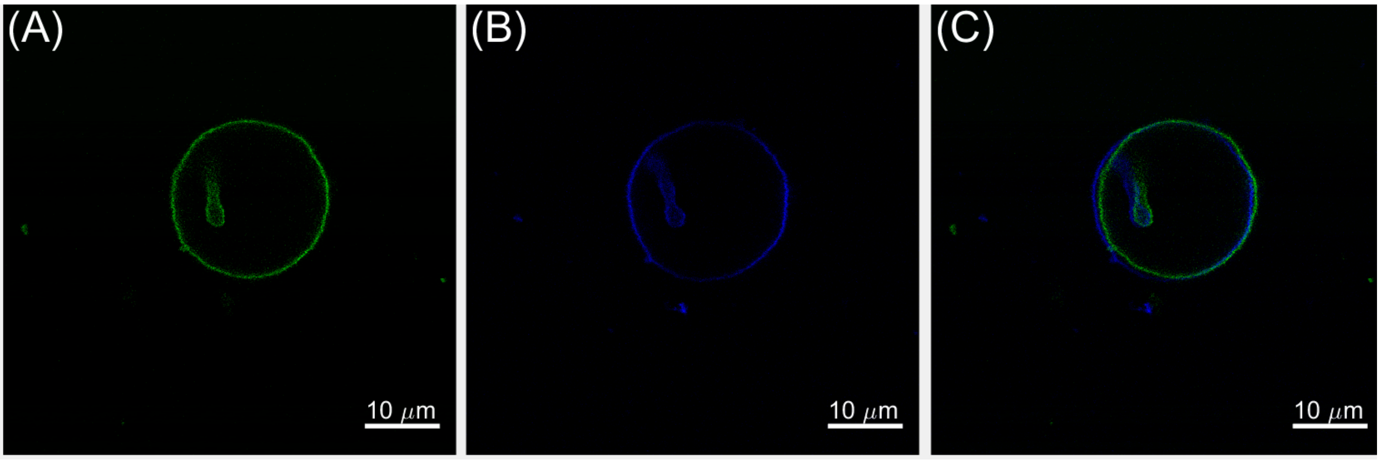

Figure S3. Fluorescence images of peptide location in lipid GUV. (A) Lipid membrane location determined using lipid probe (green). (B) Peptide location using TAMRA probe. (C) Overlay of the images.

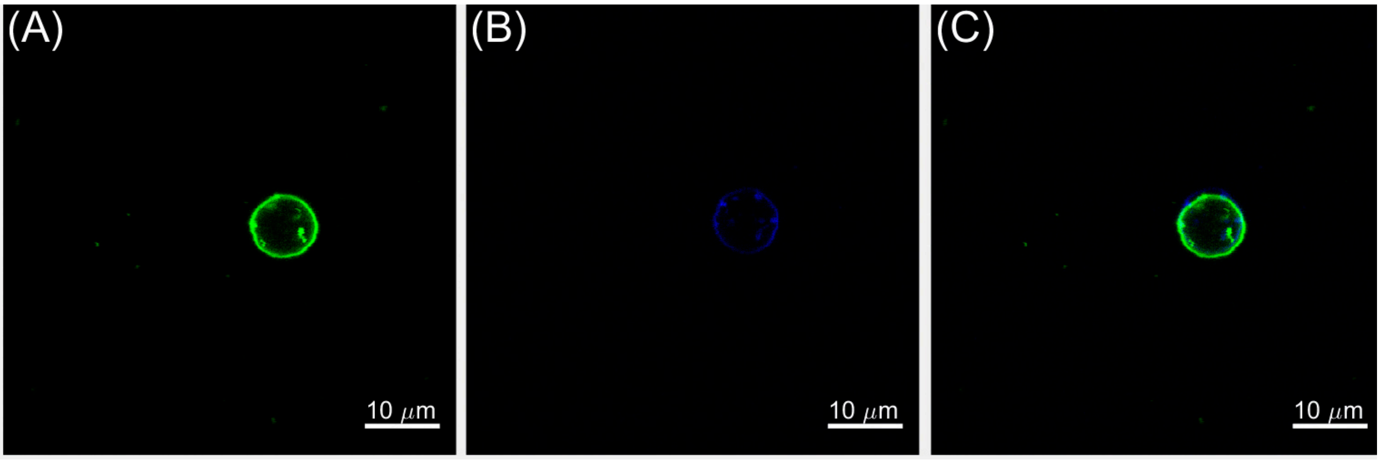

Figure S4. Fluorescence images of peptide location in lipid GUV. (A) Lipid membrane location determined using lipid probe (green). (B) Peptide location using TAMRA probe. (C) Overlay of the images.

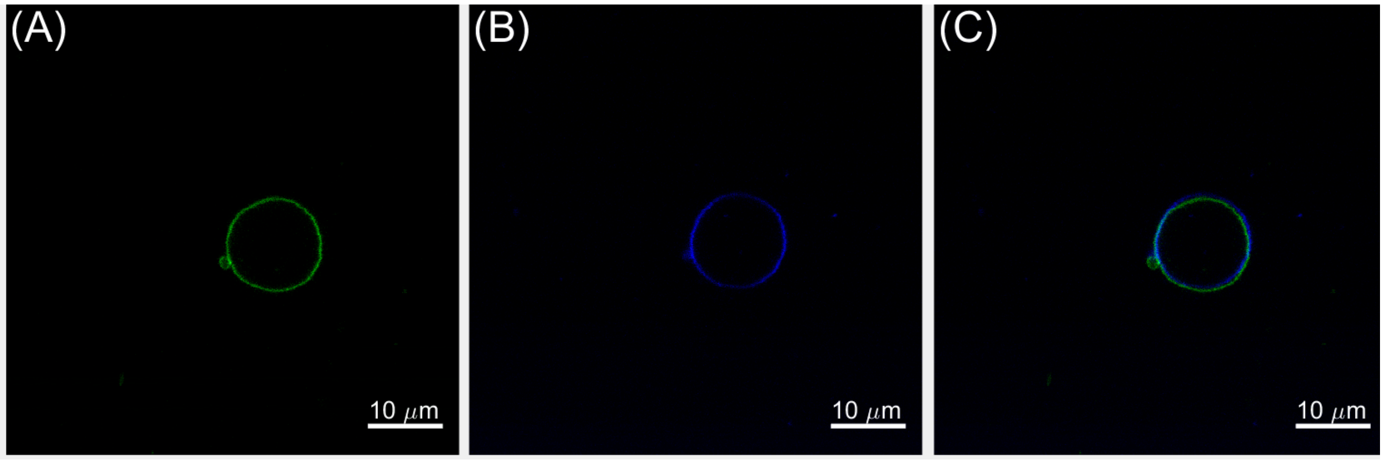

Figure S5. Fluorescence images of peptide location in lipid GUV. (A) Lipid membrane location determined using lipid probe (green). (B) Peptide location using TAMRA probe. (C) Overlay of the images.

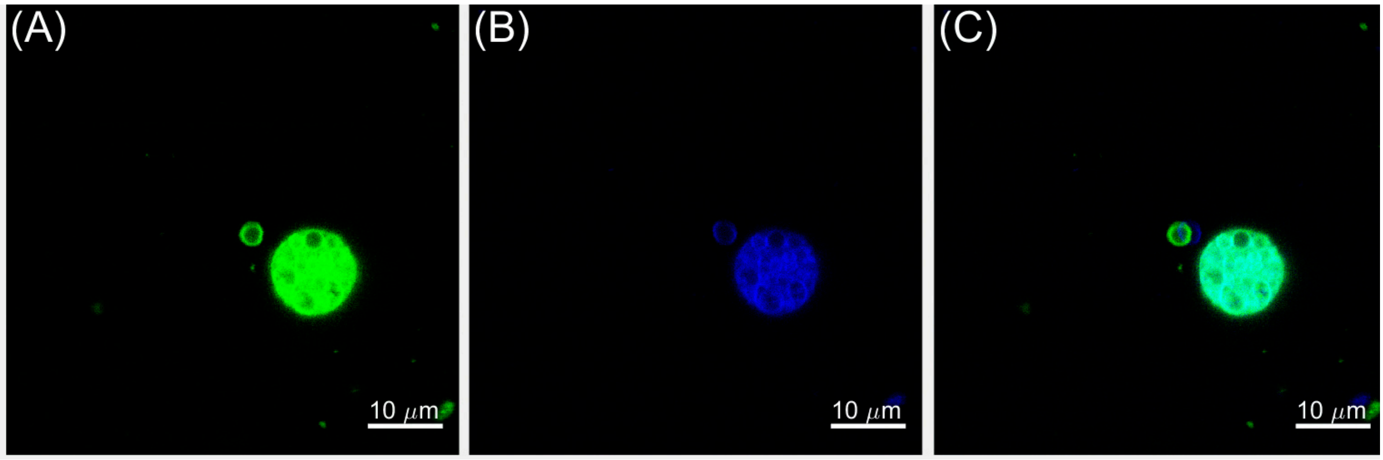

Figure S6. Fluorescence images of peptide location in lipid GUV. (A) Lipid membrane location determined using lipid probe (green). (B) Peptide location using TAMRA probe. (C) Overlay of the images.

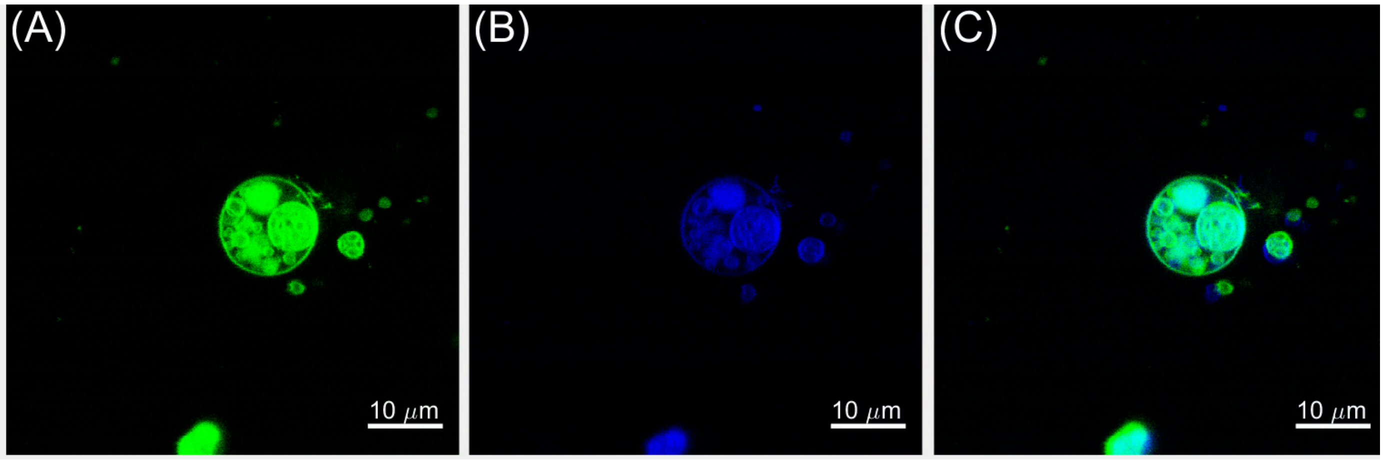

Figure S7. Fluorescence images of peptide location in lipid GUV. (A) Lipid membrane location determined using lipid probe (green). (B) Peptide location using TAMRA probe. (C) Overlay of the images.

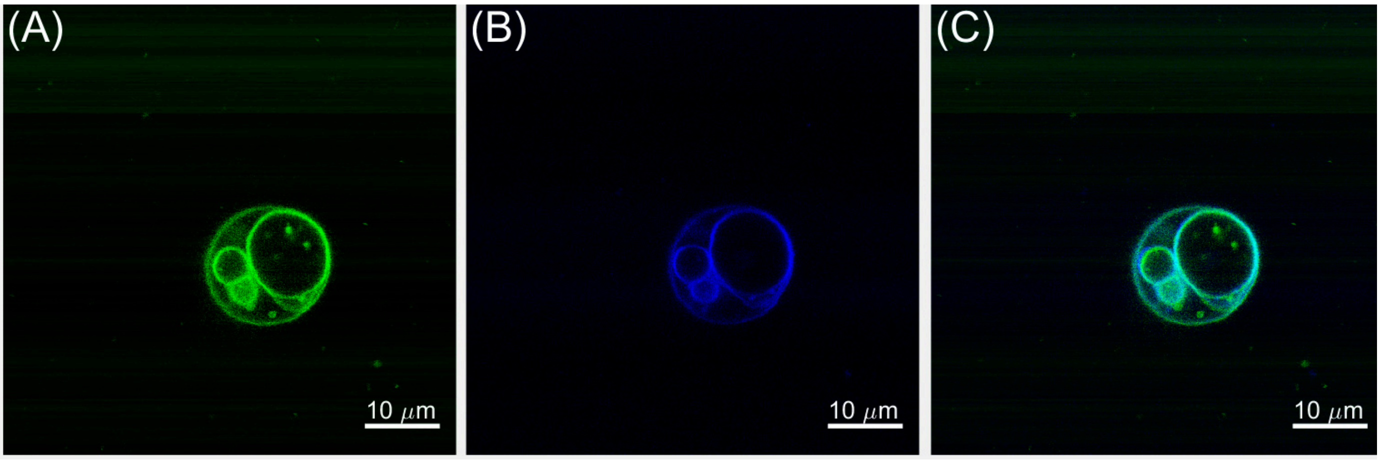

Figure S8. Fluorescence images of peptide location in lipid GUV. (A) Lipid membrane location determined using lipid probe (green). (B) Peptide location using TAMRA probe. (C) Overlay of the images.

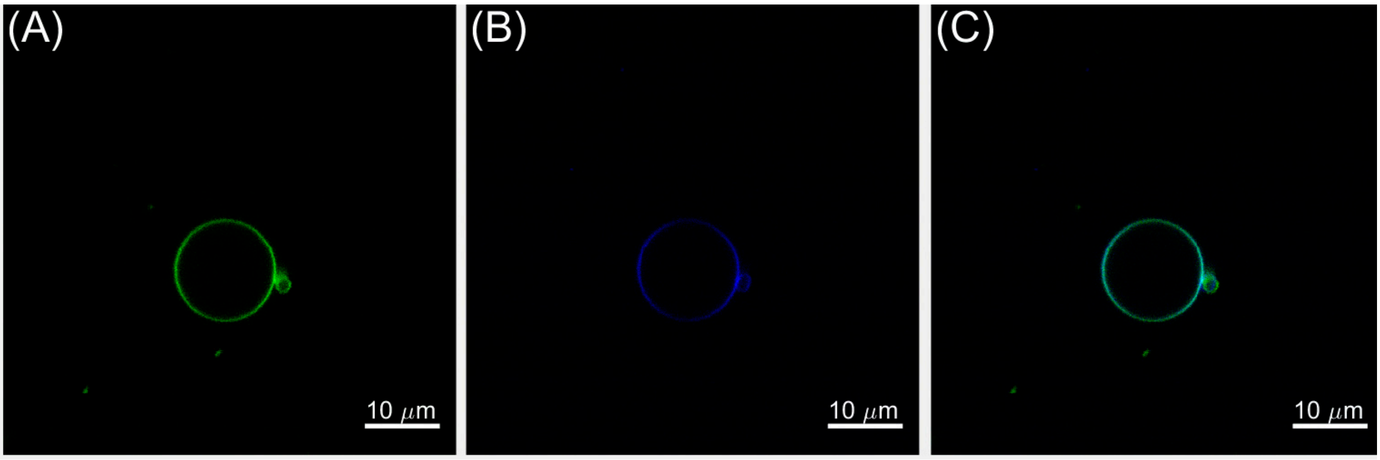

Figure S9. Fluorescence images of peptide location in lipid GUV. (A) Lipid membrane location determined using lipid probe (green). (B) Peptide location using TAMRA probe. (C) Overlay of the images.

3. Peptide incorporation into planar POPC bilayer

In order to determine peptide docking in the bilayer, several simulations were performed with various placement of the investigated peptide on the bilayer. Each system consisted of POPC lipid bilayer (100 lipids per leaflet) and was hydrated with two 40 Å water slabs. In total, seven simulations were performed, from which only three resulted in successful incorporation of the investigated peptide. The following structures of peptides were used: βA-40 and βA-42 [1]. TAMRA addition was modelled using Scigress and parameterized based on quantum calculation (single-point calculations) [2] and added to the βA-40 structure. A neutral bilayer system was chose, as it was suggested, that Aβ peptides could bind to neutral phosphatidylcholine membrane, but only in the lamellar gel phase [3].

For the βA-40 system, two approaches were tested. Two regions were identified as possible sites for incorporation, both of them showing mild hydrophobicity. In successful one, peptide was placed on bilayer by its three strong hydrophobic amino-acids (*PHE-19, PHE-20, SER-26*). As a result, after 62.6 ns, the simulation was stopped, as it was observed, that peptide incorporated in the bilayer by this region and took C-like shape on the bilayer (as presented in Supplementary Figure S10).

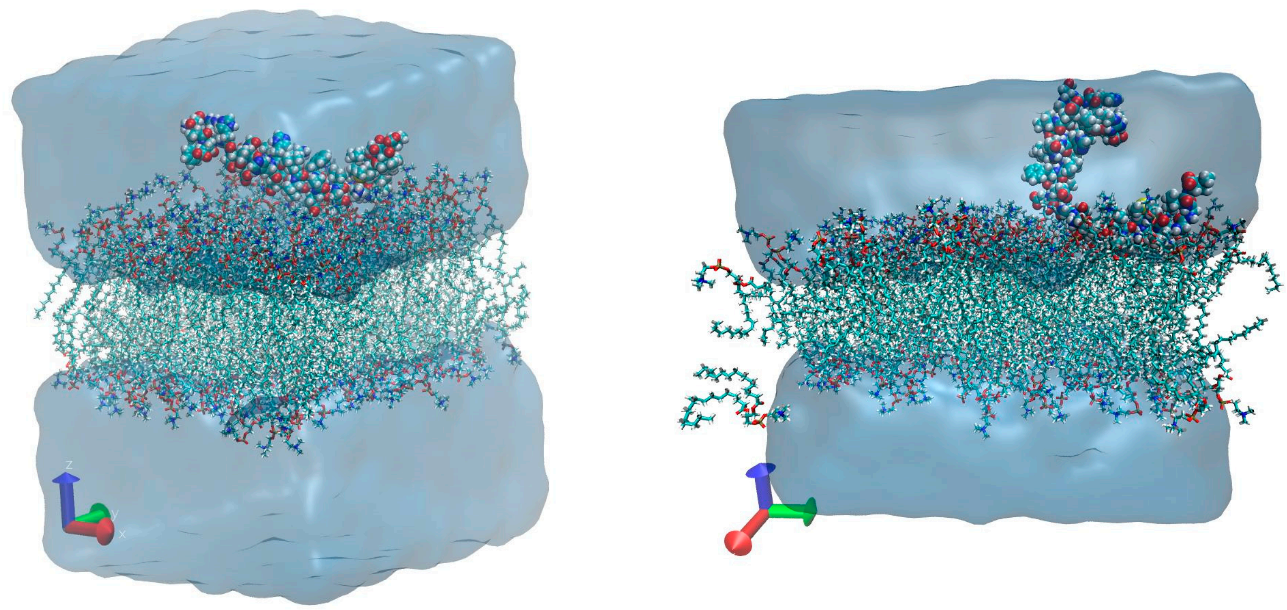

Figure S10. Successful incorporation of βA-40 into POPC bilayer system. (left) initial state, (right) final state.

In another failed approach, the peptide was pre-incorporated by mildly hydrophobic terminal region (*GLY-38, VAL-39 and VAL-40*). While initially, slight incorporation was observed, eventually, the peptide detached from the bilayer. The initial system is presented in Supplementary Figure S11.

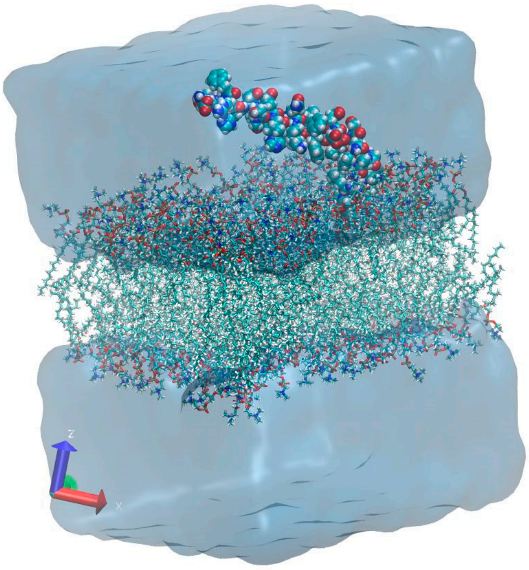

Figure S11. Initial states of failed incorporation of βA-40 into POPC bilayer system.

For the βA-42 system, three approaches were tested. Similar to βA-40, two regions were identified as possible sites for incorporation. However, successful approach was obtained when successfully incorporated βA-40 was used as a scaffold. With the modification of the peptide to add two missing amino acids a stable system configuration was achieved. Such configuration resulted in stable incorporation of the βA-42 in the bilayer, as shown in Supplementary Figure S12. The simulation was stopped after 46 ns, since system was stable. Free βA-42 monomers were reported to incorporate into bilayer less readily compared to βA-40 which might be a reason for failures in incorporation in other attempts [4].

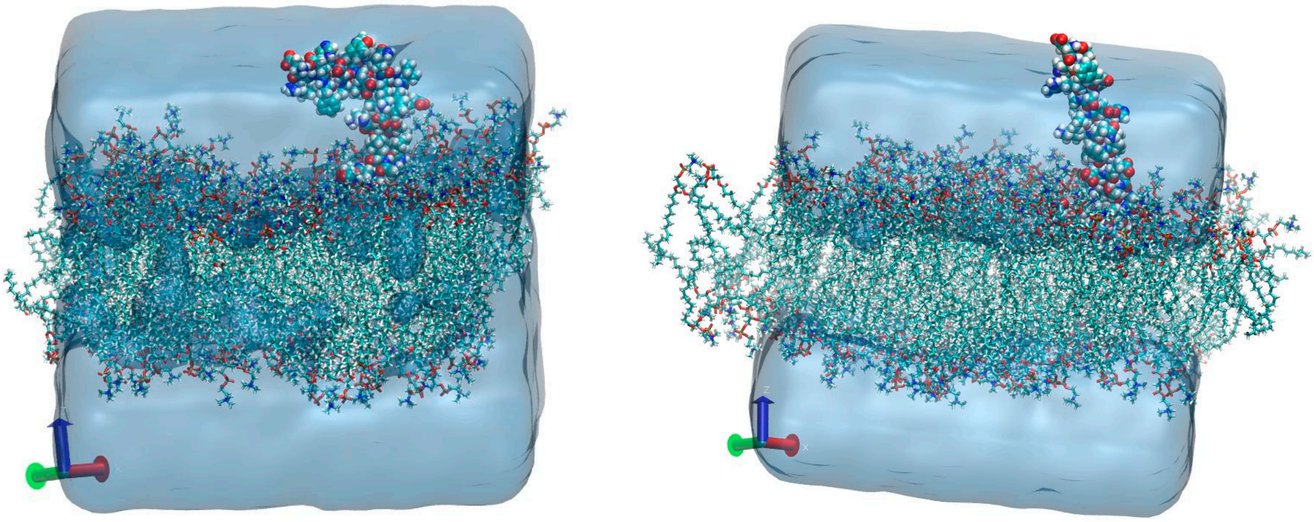

Figure S12. Successful incorporation of βA-42 into POPC bilayer system. (left) initial state, (right) final state.

Two other configurations were tested. In the first configuration, the peptide was placed in parallel on the bilayer. However, no incorporation was observed. In second approach peptide was placed on bilayer by its three strong hydrophobic amino-acids (*PHE-19, PHE-20, SER-26*). However, contrary to βA-40, incorporation wasn't observed in this case. The initial states of the system are presented in Supplementary Figure S13.

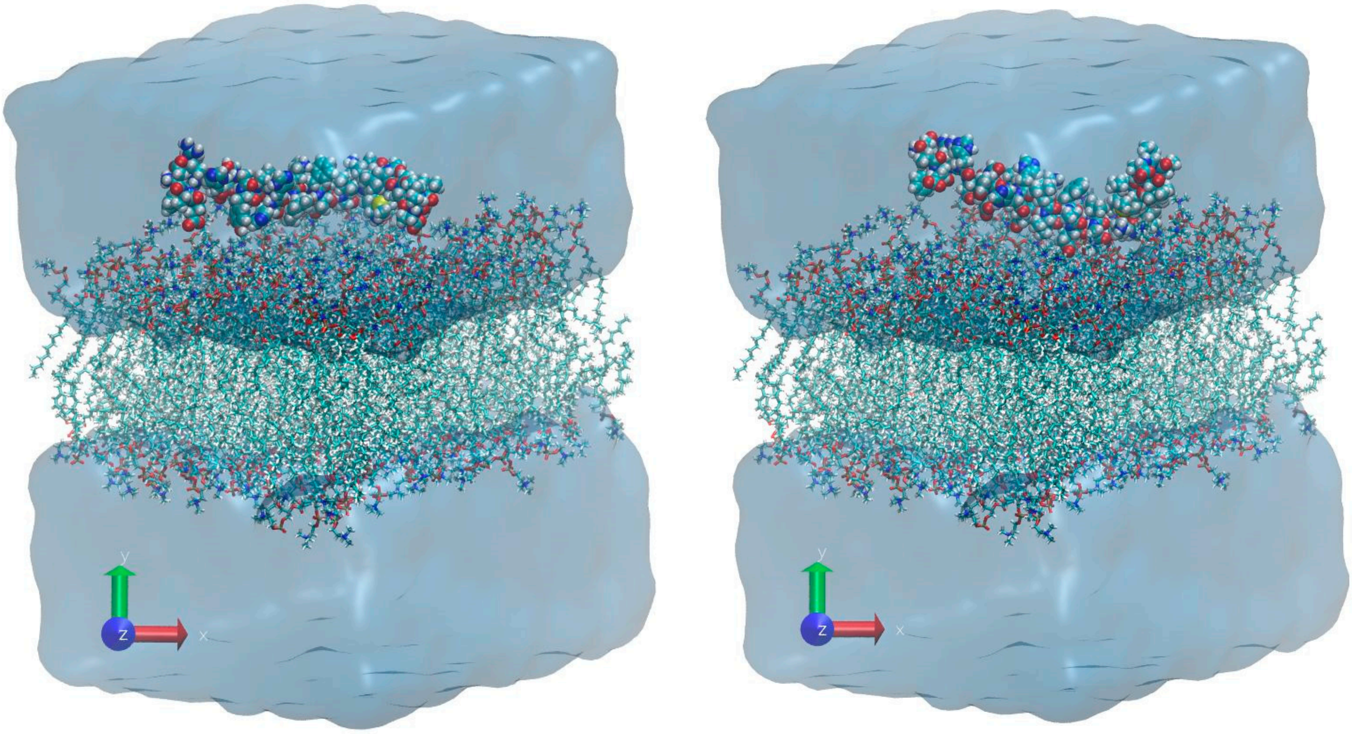

Figure S13. Initial states of failed incorporation of βA-42 into POPC bilayer system. (left) Peptide was placed parallel to the bilayer. (right) Peptide was placed by mildly hydrophobic site to the bilayer.

For the βA-40-TAMRA system, two approaches were tested. A successful approach was obtained when successfully incorporated βA-40 was used with modification of the peptide to add the fluorophore part. Such a configuration resulted in stable incorporation of the βA-40-TAMRA in the bilayer, as shown in Supplementary Figure S14. The simulation was stopped after 51 ns, since the system was stable.

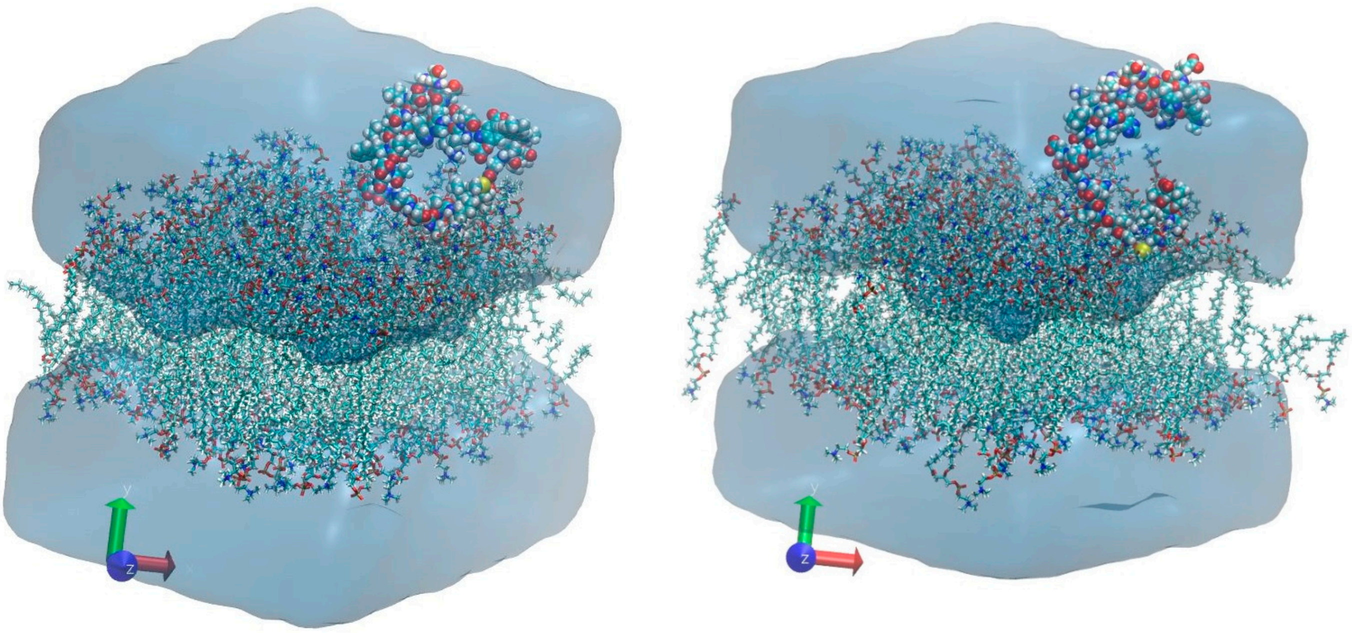

Figure S14. Successful incorporation of βA-40-TAMRA into POPC bilayer system. (left) initial state, (right) final state.

In another, failed approach, the peptide was pre-incorporated by mildly hydrophobic terminal region (*GLY-38*, *VAL-39* and *VAL-40*). However, no incorporation was observed. The initial state is presented in Supplementary Figure S15.

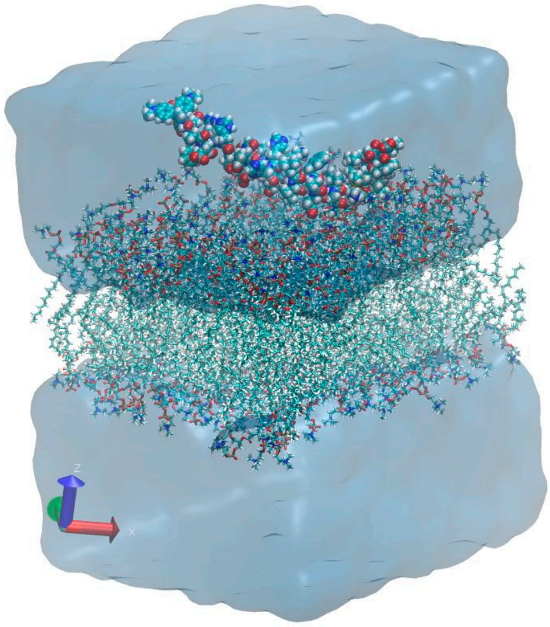

Figure S15. Initial states of failed incorporation of βA-40-TAMRA into POPC bilayer system.

4. System setup for POPC vesicles with incorporated peptides

All full-atomic simulation simulations were performed with the NAMD software [5] and united-atom CHARMM36 force field under NPT conditions. POPC vesicle system was adopted from other work [6]. The vesicle's radius was equal to 10nm. A united atom chain model was used for POPC lipids [7]. During system preparation, the aim was to achieve 10m% of peptides in the vesicles (which correspond to peptide concentration in experiment). There are 3637 lipid molecules in the vesicle system, which results in ~23 peptides if 10m% concentration is achieved. The system was hydrated with TIP3P water molecules giving a final simulation box of 30 nm3. Three-dimensional periodic boundary conditions were applied in the simulations.

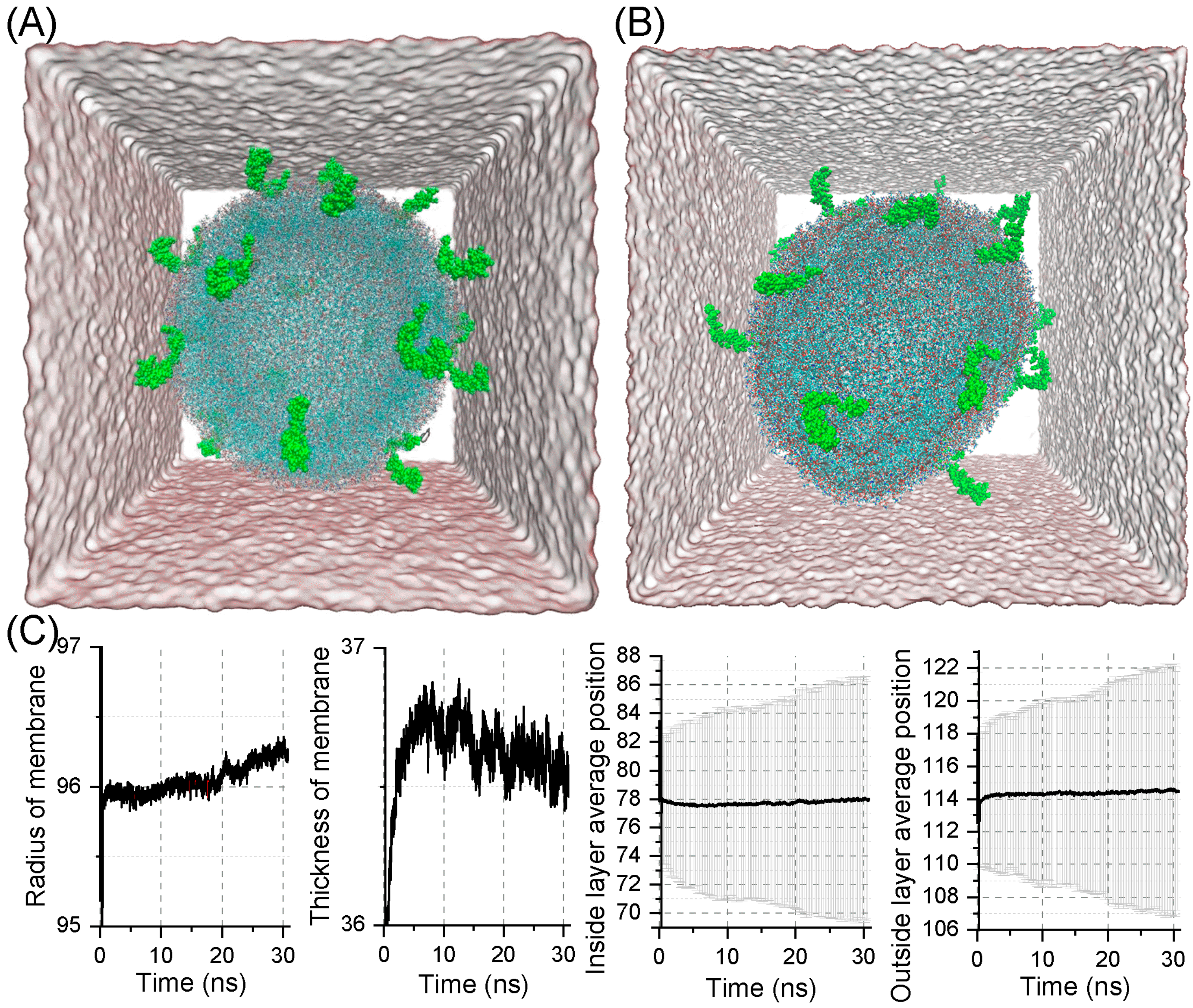

Figure S16. System setup of POPC vesicle with incorporated **Aβ-40** peptide. (A) Snapshot of initial system setup. (B) Snapshot of the system in final simulation step. (C) Details of equilibration process. Four parameters - radius of the vesicle, thickness of the membrane and inner/outer membrane position - are presented in function of simulation time.

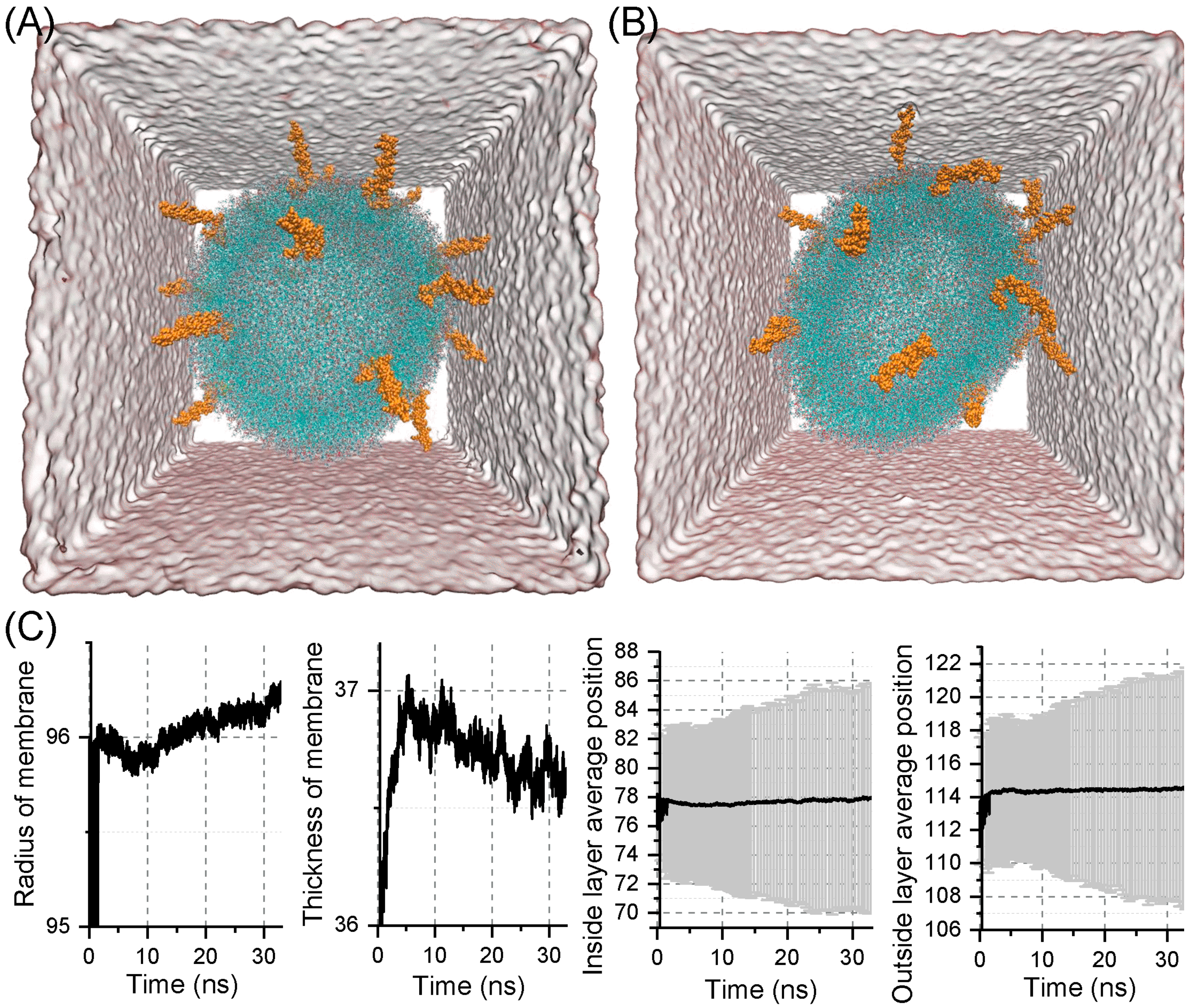

Figure S17. System setup of POPC vesicle with incorporated **Aβ-42** peptide. (A) Snapshot of initial system setup. (B) Snapshot of the system in final simulation step. (C) Details of equilibration process. Four parameters - radius of the vesicle, thickness of the membrane and inner/outer membrane position - are presented in function of simulation time.

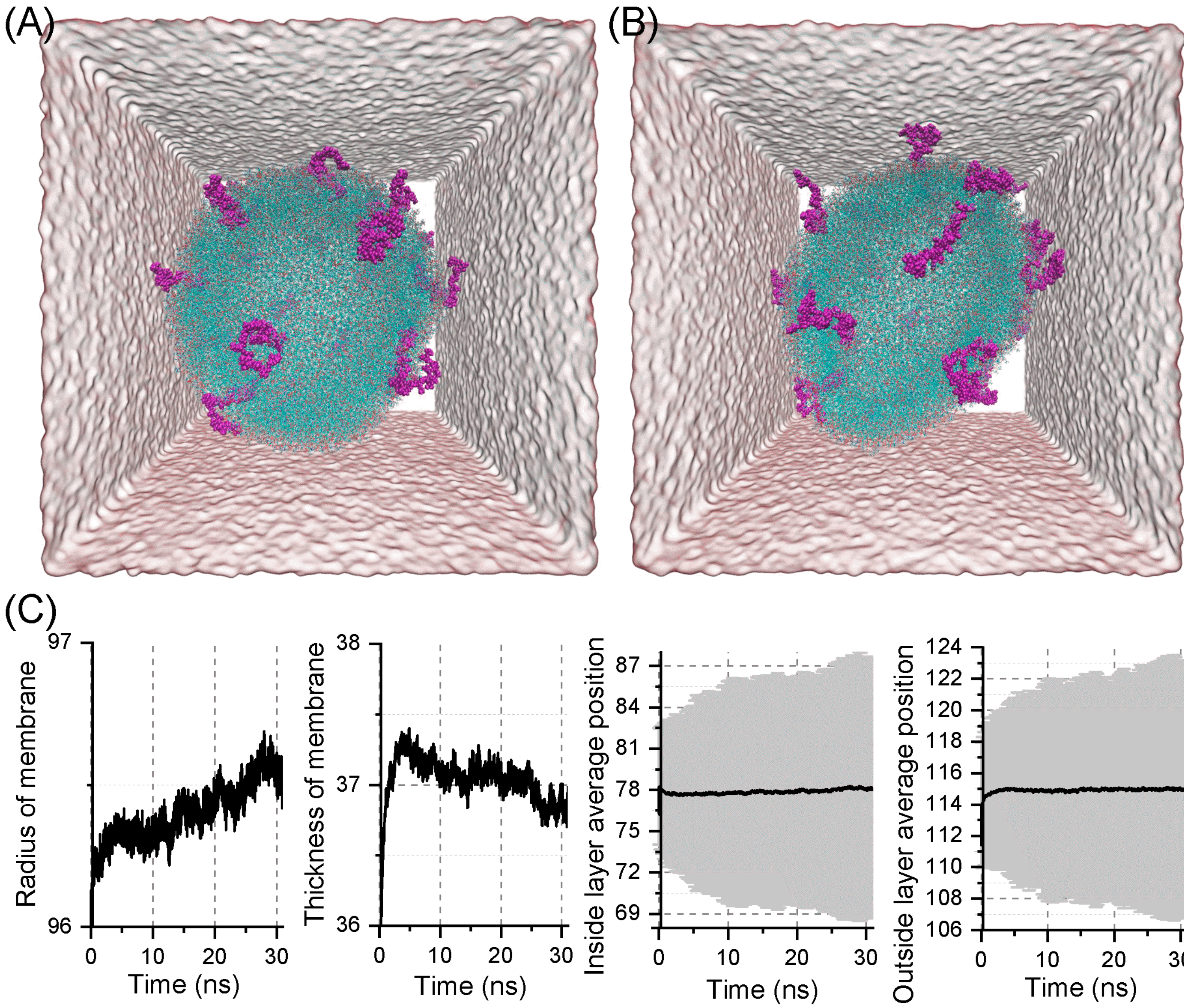

Figure S18. System setup of POPC vesicle with incorporated **Aβ-40-TAMRA** peptide. (A) Snapshot of initial system setup. (B) Snapshot of the system in final simulation step. (C) Details of equilibration process. Four parameters - radius of the vesicle, thickness of the membrane and inner/outer membrane position - are presented in function of simulation time.

Literature

1. Crescenzi, O.; Tomaselli, S.; Guerrini, R.; Salvadori, S.; D'Ursi, A.M.; Temussi, P.A.; Picone, D. Solution structure of the Alzheimer amyloid beta-peptide (1-42) in an apolar microenvironment - Similarity with a virus fusion domain. *Eur J Biochem* **2002**, *269*, 5642-5648, doi:10.1046/j.1432-1033.2002.03271.x.
2. Barucha-Kraszewska, J.; Kraszewski, S.; Ramseyer, C. Will C-Laurdan Dethrone Laurdan in Fluorescent Solvent Relaxation Techniques for Lipid Membrane Studies? *Langmuir : the ACS journal of surfaces and colloids* **2013**, *29*, 1174-1182, doi:10.1021/la304235r.
3. Yoda, M.; Miura, T.; Takeuchi, H. Non-electrostatic binding and self-association of amyloid beta-peptide on the surface of tightly packed phosphatidylcholine membranes. *Biochemical and biophysical research communications* **2008**, *376*, 56-59, doi:10.1016/j.bbrc.2008.08.093.
4. Chang, C.C.; Edwald, E.; Veatch, S.; Steel, D.G.; Gafni, A. Interactions of amyloid-beta peptides on lipid bilayer studied by single molecule imaging and tracking. *Biochimica et biophysica acta. Biomembranes* **2018**, 10.1016/j.bbamem.2018.03.017, doi:10.1016/j.bbamem.2018.03.017.
5. Philips, J.C.; Braun, R.; Wang, W.; Gumbart, J.; Tajkhorsid, E.; Villa, E.; Chipot, C.; Skeel, R.D.; Kalé, L.; Schulten, K. Scalable molecular dynamics with NAMD. *Journal of Computational Chemistry* **2005**, *26*, 1781-1802.
6. Drabik, D.; Chodaczek, G.; Kraszewski, S.; Langner, M. Mechanical Properties Determination of DMPC, DPPC, DSPC, and HSPC Solid-Ordered Bilayers. *Langmuir : the ACS journal of surfaces and colloids* **2020**, *36*, 3826-3835, doi:10.1021/acs.langmuir.0c00475.
7. Lee, S.; Tran, A.; Allsopp, M.; Lim, J.B.; Hénin, J.; Klauda, J.B. CHARMM36 United Atom Chain Model for Lipids and Surfactants. *J. Phys. Chem. B* **2014**, *118*, 547-556.
